# *Hericium erinaceus* and *Coriolus versicolor* Modulate Molecular and Biochemical Changes after Traumatic Brain Injury

**DOI:** 10.3390/antiox10060898

**Published:** 2021-06-02

**Authors:** Ramona D’Amico, Angela Trovato Salinaro, Roberta Fusco, Marika Cordaro, Daniela Impellizzeri, Maria Scuto, Maria Laura Ontario, Gianluigi Lo Dico, Salvatore Cuzzocrea, Rosanna Di Paola, Rosalba Siracusa, Vittorio Calabrese

**Affiliations:** 1 Department of Chemical, Biological, Pharmaceutical and Environmental Sciences, University of Messina, 98166 Messina, Italy; rdamico@unime.it (R.D.); rfusco@unime.it (R.F.); dimpellizzeri@unime.it (D.I.); rsiracusa@unime.it (R.S.); 2 Department of Biomedical and Biotechnological Sciences, University of Catania, 95125 Catania, Italy; trovato@unict.it (A.T.S.); mary-amir@hotmail.it (M.S.); marialaura.ontario@ontariosrl.it (M.L.O.); gigilodico@gmail.com (G.L.D.); calabres@unict.it (V.C.); 3 Department of Biomedical, Dental and Morphological and Functional Imaging University of Messina, Via Consolare Valeria, 98125 Messina, Italy; marika.cordaro@unime.it; 4 Department of Pharmacological and Physiological Science, Saint Louis University School of Medicine, Saint Louis, MO 63104, USA

**Keywords:** brain trauma, neurodegeneration, neuroinflammation, oxidative stress, mushrooms

## Abstract

Traumatic brain injury (TBI) is a major health and socioeconomic problem affecting the world. This condition results from the application of external physical force to the brain which leads to transient or permanent structural and functional impairments. TBI has been shown to be a risk factor for neurodegeneration which can lead to Parkinson’s disease (PD) for example. In this study, we wanted to explore the development of PD-related pathology in the context of an experimental model of TBI and the potential ability of *Coriolus versicolor* and *Hericium erinaceus* to prevent neurodegenerative processes. Traumatic brain injury was induced in mice by controlled cortical impact. Behavioral tests were performed at various times: the animals were sacrificed 30 days after the impact and the brain was processed for Western blot and immunohistochemical analyzes. After the head injury, a significant decrease in the expression of tyrosine hydroxylase and the dopamine transporter in the substantia nigra was observed, as well as significant behavioral alterations that were instead restored following daily oral treatment with *Hericium erinaceus* and *Coriolus versicolor*. Furthermore, a strong increase in neuroinflammation and oxidative stress emerged in the vehicle groups. Treatment with *Hericium erinaceus* and *Coriolus versicolor* was able to prevent both the neuroinflammatory and oxidative processes typical of PD. This study suggests that PD-related molecular events may be triggered on TBI and that nutritional fungi such as *Hericium erinaceus* and *Coriolus versicolor* may be important in redox stress response mechanisms and neuroprotection, preventing the progression of neurodegenerative diseases such as PD.

## 1. Introduction

Traumatic brain injury (TBI) can be divided into three phases: acute (0 to 1 week), post-acute (1 to 4 weeks) and chronic (4 weeks to years). During the first phase, cell necrosis occurs due to the direct impact of an external force on the brain tissue. Cell death subsequently occurs due to axonal changes and inflammation. The post-acute phase can be characterized by a reduction in inflammation, neuronal remodeling but also by an increase in chronic pathology [1]. The last phase includes incorrect protein folding, prolonged and persistent inflammation and neurodegeneration. TBI therefore represents the non-genetic risk factor for several neurodegenerative diseases, including Alzheimer’s disease (AD), amyotrophic lateral sclerosis (ALS) and Parkinson’s disease (PD) [2,3,4]. Prospective cohort studies were performed in order to determine the relationship between TBI and PD [3,5,6]. Additionally, in a recent study, researchers analyzed the medical records of 325,870 veterans, many of whom (about half) had suffered mild, moderate or severe head injury. At the start of the study, no veteran had a diagnosis of PD. After 12 years, however, 1462 veterans were diagnosed with PD and among them 949 had suffered a brain injury. Therefore, after adjusting the data based on the patient’s age, medical condition, and other factors, the researchers showed that veterans with mild TBI have a 56% risk of developing PD later in life, while this percentage rises to 83% with the increase in the severity of the brain injury [7].

To date, there is no real treatment for PD or for the long-term consequences of TBI but only palliative care. Therefore, understanding the mechanistic relationship between TBI and PD is important for studies aimed at discovering compounds that are capable of slowing or preventing PD in at-risk populations. 

The mechanisms involved in the progression of neurodegenerative diseases such as PD are various. Among the most common we have mitochondrial dysfunction, the accumulation of proteins such as α-synuclein, oxidative stress and neuroinflammation. TBI is known to cause a breakdown of the blood-brain barrier and damage to the neurovascular unit leading to disruption of homeostasis, excitotoxicity, oxidative stress and inflammation, all factors that over time lead to neurodegeneration [8,9,10,11,12,13,14,15]. These events can continue even after the initial injury creating a favorable environment for the development of PD. Therefore, we hypothesize that compounds with anti-inflammatory and anti-oxidant activity may be a good strategy for the prevention of TBI-related neurodegenerative diseases. 

Mushrooms contain a wide range of bioactive compounds that encourage their use both in the maintenance of human health and in the prevention of diseases often related to the central nervous system. In particular *Hericium erinaceus* is among the most characterized medicinal mushrooms and with a focus on the nervous system [16]. An increasing number of anti-neuroinflammatory activities of this mushroom have been reported in recent years. In particular, in a study of 15-month-old mice given a diet rich in fat and sucrose it was found that it improves spatial learning abilities and reduces the expression of tumor necrosis factor-alpha (TNF-α) and interleukin-1β (IL-1β) mRNA in the hippocampus therefore having an anti-neuroinflammatory effect [17]. In another study in which the mushroom was administered for 30 days, the ability to reduce the activation of astrocytes and microglia in the cerebral cortex and hippocampus of AD APPswe/PS1dE9 transgenic mice was shown. Furthermore, in an AD mouse model it has been shown that the anti-inflammatory activity of *Hericium erinaceus* also reduces the deposition of Aβ, improves the expression of nerve growth factor (NGF) and the neurogenesis of the hippocampus [18]. The anti-neuroinflammatory activity of *Hericium erinaceus* has been demonstrated in models of Alzheimer’s and other models of neurodegenerative disorders such as epilepsy [19]. It has also been observed that *Hericium erinaceus* in addition to having anti-inflammatory activity it also has an anti-oxidant action as it is capable of acting on the NF-E2-related factor 2 (Nrf2)/heme oxygenase 1 (HO-1) pathway [20,21]. *Hericium erinaceus* could therefore be considered an excellent neuroprotector. 

Another mushroom extensively studied in recent years and which has been shown to have a neuroprotective effect is *Coriolus versicolor*. Recent studies have suggested that this mushroom has anticancer, anti-inflammatory, antioxidant, antibacterial and immunomodulatory properties [22,23,24]. Like *Hericium erinaceus, Coriolus versicolor* has also been shown to improve spatial memory in a mouse model of AD by increasing antioxidant activity (superoxide dismutase (SOD) and catalase (CAT)) and inhibition of pro-inflammatory cytokines (IL-1β, interleukin-6 (IL-6) and TNF-α) [25]. Furthermore, treatment with *Coriolus versicolor* has been shown to promote the up-regulation of the anti-inflammatory mediator lipoxin A4 and the increase in the levels of proteins involved in the cellular stress response such as heat shock protein 70 (Hsp70), HO-1 and thioredoxin (Trx) in the cortex and hippocampus [26]. 

On the basis of the data in the literature, the aim of our study was to evaluate how treatment with *Hericium erinaceus, Coriolus versicolor* and with their association was able to act on the molecular, biochemical and cellular changes that occur in the brain after trauma and that increase the risk of developing PD. 

## 2. Materials and Methods

### 2.1. Animals

Male CD1 mice (25 to 30 g, Envigo, Casatenovo, Italy) were used for all studies. Mice were housed in individual cages (five per cage) and maintained under a 12:12 h light/dark cycle at 21 ± 1 °C and 50 ± 5% humidity. Standard laboratory diet and tap water were available ad libitum. The Review Board for the care of animals of the University of Messina approved the study (ethical protocol code: n° 211/2021-PR). We respected the legislation for the protection of laboratory animals (D.Lgs 2014/26 and EU Directive 2010/63).

### 2.2. Controlled Cortical Impact (CCI) Experimental TBI

TBI was induced in mice by a controlled cortical impact (CCI) as previously described [27,28]. A craniotomy was made and on the exposed cortex a cortical contusion was produced using the controlled impactor device Impact OneTM Stereotaxic impactor for CCI (Leica, Milan, Italy) (tip diameter: 4 mm; cortical contusion depth: 3 mm; impact velocity: 1.5 m/s). Sham mice underwent the identical surgical procedure but were not injured.

#### Experimental Design

All animals were randomized in the indicated groups (10 mice for each group): 

Sham + vehicle group: mice were subjected to the surgical procedures as above except that the impact was not applied and animals were treated o.s. with vehicle;

Sham + *Hericium erinaceus* (H): mice were subjected to the surgical procedures as above except that the impact was not applied and animals were treated o.s. with H (data not shown);

Sham + *Coriolus versicolor* (C): mice were subjected to the surgical procedures as above except that the impact was not applied and animals were treated o.s. with C (data not shown);

Sham + *Hericium erinaceus* + *Coriolus versicolor* (H + C): mice were subjected to the surgical procedures as above except that the impact was not applied and animals were treated o.s. with H + C (data not shown);

TBI + vehicle group: mice were subjected to CCI plus administration of vehicle (saline); 

TBI + *Hericium erinaceus* (H): As for the TBI + vehicle group but H was administered o.s. at 200 mg/kg in saline for 30 days after TBI; 

TBI + *Coriolus versicolor* (C): As for the TBI + vehicle group but C was administered o.s 200 mg/kg dissolved in saline for 30 days after TBI; 

TBI + *Hericium erinaceus* + *Coriolus versicolor* (H + C): As for the TBI + vehicle group but H (200 mg/kg) + C (200 mg/kg) was administered o.s for 30 days after TBI.

*H. erinaceus* and *C. versicolor* biomasses including mycelium and primordia of the respective mushroom, generous provided by Mycology Research Laboratories Ltd. (MRL, Luton, UK), as the product commercially existing, were used for investigates. Optimal quantity (200 mg/kg) was selected according to the dose used in clinical trials with cancer or HPV patients (3 g/day) [29], a regimen also confirmed by studies in rat [17,26].

Mice were sacrificed 30 days after surgical procedures brain was collected for histological and biochemical investigation. In addition, over the 30-days experimental period behavioral changes at 1, 7, 14, and 30 days were evaluated.

### 2.3. Chemical Reagents

All reagents used are HPLC grade. Ultrapure deionized water, with resistivity of 18.2 megaohm-cm was obtained from a Milli-Q^®^ Integral water purification system with Q-pod (Millipore, Bedford, MA, USA). Formic acid was purchased from Carlo Erba (Milan, Italy). Ethanol, acetonitrile and methanol from Merck (Darmstadt, Germany). Ammonium acetate, acetone and 2-propanol were purchased from Carlo Erba (Milan, Italy). The SPE cartridges Waters Oasis HLB (200 mg) from Waters S.p.A. (Milan, Italy). Standard solutions of gallic acid, ascorbic acid, vanillic acid, caffeic acid, chlorogenic acid, catechin, epicatechin, vanillic acid, coumaric acid and hydroxybenzoic acid were purchased from Extrasynthese (Genay Cedex, France); quercetin, rutin, apigenin, luteolin, ferulic acid, kaempferol, palmitic acid, stearic acid, oleic acid, linoleic acid, glucose, *N*,*O*-bis(trimethylsilyl) trifluoroacetamide (BSTFA), trimethylchlorosilane (TMCS) and pyridine were acquired from Sigma-Aldrich S.r.l. (Milan, Italy).

### 2.4. Sample Pretreatment

The sample of C, in the form of tablets, was subjected to crushing in a mortar and passed through a sieve with a mesh size of 20 µm. The H sample in powder form was only sieved with a mesh size of 20 µm.

### 2.5. Extraction Method

Two 0.1 g aliquots of C powder and 0.1 g aliquots of H powder were solubilized in 10 mL of ethanol and 10 mL of acetonitrile, respectively, in orbital shaker incubator (Professional 3500 Orbital Shaker, VWR International Srl, Milan, Italy) at room temperature for 24 h. The extracts were filtered in a syringe filter (Thermo Fisher Scientific Inc., Waltham, MA, USA) of size 0.45 μm. Solubilized samples were then passed through SPE cartridges, previously conditioned with 4 mL of methanol and 2 mL of deionized water, followed by washing with 2 × 10 mL of water and eluted with 1 mL of ethanol for Liquid Chromatography-Orbitrap-Mass Spectrometry (LC-Orbitrap-MS) and 1 mL of acetonitrile for**** Gas Chromatography-Tandem Mass Spectrometry (GC-MS/MS) analyses. The ethanol extract was reduced in small volumes in an evaporator under a current of nitrogen and taken up with 1 mL of mobile phase.

### 2.6. GC-MS/MS

1 mL of the acetonitrile extract got was mixed with 30 μL of N,O-bis(trimethylsilyl) trifluoroacetamide (BSTFA), 10 μL of 1% trimethylchlorosilane (TMCS) reagent, and about 15 μL of pyridine inside a tube. Then, the tube was positioned into a water bath at 70 °C for 2 h; the mixture was ready for GC-MS/MS analysis. The derivatization was carried out in order to improve the chromatography and the identification of the peaks. The extract was analyzed with a Thermo Scientific TSQ-Quantum XLS Triple Quadrupole GC-MS/MS gas chromatography mass spectrometry system (Thermo Fisher Scientific, Waltham, MA, USA). MS acquisition time of 15.52 min for analysis of C and 15.50 min for H in full scan mode (50–1000 amu) equipped of a TR-5MS column 5% phenyl methyl siloxane, 30 m × 250 μm × 0.25 μm; scan time 0.272; Q1 peak width 0.70 eV in positive and negative polarity. Furnace temperatures have been set: initial holding time 100 °C and rate of 14 °C/min for 1 min at 330 °C; 5 μL injected in programmed temperature vaporization (PTV).

### 2.7. LC–Orbitrap-MS

A Q-Exactive Plus Hybrid Quadrupole-Orbitrap^TM^ Mass Spectrometer (Thermo Fisher Scientific, Waltham, MA, USA) equipped with a Heated ElectroSpray Ionization (HESI) system was used in positive and negative polarity manners for the identification of the precise masses. The operation parameters were as follows: cover gas flow amount, 35 (random units); aux gas flow amount, 10 (random units); spray voltage, 3.50 kV; capillary temperature, 300 °C; tube lens voltage, 55 V; heater temperature, 305 °C; scan mode: full scan; scan range (*m*/*z*) 100–1000; microscans, 1 *m/z*; positive resolution: 70,000; fourier transform (FT) automatic gain control (AGC) target: 3 × 10; negative resolution: 35,000; automatic gain control (AGC) target: 1 × 106; maximum IT: 100 ms. The chromatographic parameters were as follows: column temperature, 30 °C; sample temperature, 6 °C; flow rate, 0.2 mL min^−1^. The autosampler sample holder temperature was maintained at 7 °C. The mobile phase consisted of eluent A: 30 mM ammonium acetate (pH 5), eluent B: methanol, eluent C: water (0.5% formic acid), and eluent D: acetonitrile/acetone/2-propanol (4:3:3). Moveable phases A and B were used to enhance the chromatographic resolution; moveable phases B, C, and D were obligatory for purification in TurboFlow^TM^ (Thermo Fisher Scientific, Waltham, MA, USA). The sample injection volume was 5 μL with injection syringe. Cyclone P column (50 mm × 0.5 m, 60 μm particle size, 60 Å pore size, Thermo Fisher Scientific, Waltham, MA, USA); a Hypersil Gold (2.1 mm × 100 mm, 1.7 μm particle size) column was working as the analytical separation column. The total run time was 10 min for analysis of C and 13 min for analysis of H. The data analysis was performed using Thermo Scientific XCalibur (Thermo Fisher Scientific, Waltham, MA, USA) version 4.0 software and Qual Browser (Thermo Fisher Scientific, Waltham, MA, USA).

### 2.8. Behavioral Testing

All animals were subjected to behavior examinations at 1, 7, 14, and 30 days post-CCI. The mice were positioned in behavior places 5 min for 2 days for acclimation earlier to the onset of behavioral analysis. Three different reliable expert observers blinded to the injury status of the animals conducted the behavioral tests. Tests are described below:

#### 2.8.1. Elevated Pluz-Maze (EPM)

The Elevated pluz-maze Test (EPMT) test was performed as described previously [30]. The EPMT apparatus (Panlab, S.L.- Harvard Apparatus, Barcelona, Spain) was employed. The trial was founded on rodents’ aversion to open areas that leads to thigmotaxis. Anxiety was denoted by an increase in the amount of time consumed in the closed arms and a diminution in the proportion of entries into the open arms. The entire number of arm entries and number of closed-arm entries were used as measures of usual activity.

#### 2.8.2. Open Field Test (OF)

Locomotor activity and anxious behavior were videotaped for 5 min using the open field test (50 × 50 cm Plexiglas box with the floor divided into 16 squares). Four squares represented the center and 12 squares along the walls represented the periphery. Each mouse was placed in the center and activity was evaluated as a line that crosses when a mouse moves its four legs from one square and enters another. The line passes and the time spent in the center have been counted and marked [31].

#### 2.8.3. Barnes Maze 

The Barnes Maze is a validated and often used test to assess spatial learning and memory in rodents. Test was performed as described previously [32]. Performance was measured by the number of errors made by the rodent and the rate of diminution in the number of errors per test was considered to represent a learning curve.

### 2.9. Histology

The brains (*n* = 5/each group) were fixed, cut and stained (Hematoxylin and Eosin, Bio-Optica, Milan, Italy). Afterward, the slices were detected under an optical microscope associated to an imaging system (AxioVision, Zeiss, Milan, Italy). Histopathologic changes of the gray matter were scored on a six-point scale as described by Campolo et al. [6]. Furthermore, the histopathologic alteretions of the midbrain were scored as described in previously study [33]. The various sections were then averaged to obtain a final score for each individual mouse. The images are representative of all animals in each group.

### 2.10. Immunohistochemical Analysis of Tyrosine Hydroxylase (TH), Dopamine Transporter (DAT)

The technique we used was performed as previously described [34]. The antibodies we investigated were incubated overnight on the brain sections: anti-TH (Millipore, Milan, Italy, 1:500 in PBS, *v*/*v*, AB152), anti-DAT (Santa Cruz Biotechnology, Segrate, Milan, Italy, 1:300 in PBS, *v*/*v*, 65G10 sc-32258). In order to assess the specificity of the antibodies, brain sections (from five mice for each group) were treated with primary antibody or secondary antibody only. The images were taken using a Zeiss microscope (AxioVision, Zeiss, Milan, Italy) and AxioVision software (version 4.7.2, AxioVision, Zeiss, Milan, Italy). The ImageJ IHC profiler plug-in (version 1.49v, Java 1.8.0_45, Wayen Rasband, U.S. National Institutes of Health, Bethesda, MD, USA) was used for densitometric analysis as described by Sawant et al. [35]. The resulting histogram profile corresponds to the intensity value of the positive pixel obtained by the computer program [36]. Immunohistochemical analyses were performed by experienced people who were unfamiliar with the treatment. 

### 2.11. Western Blot Analysis for GFAP, Iba-1, IkB-α, NF-kB, Nrf2, HO-1, Hsp70, γ-GCs, Trx, α-syn, Bax, Bcl-2

Western blot analysis was performed on brains of five mice for each group with the protocol that we previously described [37]. The levels of glial fibrillary acidic protein (GFAP), ionized calcium-binding adapter molecule 1 (Iba-1), nuclear factor of kappa light polypeptide gene enhancer in B-cells inhibitor, alpha (IkB-α), HO-1, Hsp70, γ-glutamylcysteine synthetase (γGCs), Trx, BCL2-associated X protein (Bax), Bcl-2 and α-synuclein (α-syn) were quantified in cytosolic, while nuclear factor kappa-light-chain-enhancer of activated B cells (NF-kB p65) and Nrf2 levels were quantified in nuclear fraction. The specific primary antibodies that have been incubated at 4 °C overnight are: anti-GFAP (1:500; Santa Cruz Biotechnology, 2E1 sc-33673), anti-Iba-1 (1:500; Santa Cruz Biotechnology, 1022-5 sc-32725), anti-IkB-α (1:500; Santa Cruz Biotechnology, H-4 sc-1643), anti-NF-kB p65 (1:500; Santa Cruz Biotechnology, F-6: sc-8008), anti-Bax (1:500; Santa Cruz Biotechnology, B-9 sc-7480), anti-Bcl-2 (1:500; Santa Cruz Biotechnology, C-2 sc-7382), anti-α-syn (1:500; Santa Cruz Biotechnology, LB509 sc-58480), anti-Nrf-2 (1:500; Santa Cruz Biotechnology, A-10 sc-365949), anti-HO-1 (1:500; Santa Cruz Biotechnology, A3 sc-136960), anti-Hsp70 (1:500; Santa Cruz Biotechnology, 3A3 sc-32239), anti-γGCs (1:500; Santa Cruz Biotechnology, H5 sc-390811), anti-Trx (1:500; Santa Cruz Biotechnology, A-5 sc-166393). Protein lysates were additionally incubated with β-actin or laminin antibody (1:5000; Santa Cruz Biotechnology, C4 sc-47778, E1 sc-376248) in order to attest that all samples had been loaded in identical amounts. The signals were imprisoned with BIORAD ChemiDocTM XRS + software (BioRad, Laboratories, Hercules, CA, USA) with the use of a chemiluminescent reagent (Super Signal West Pico chemiluminescent substrate, Pierce, Chemical, Dallas, TX, USA). The comparative expression of the bands was successively normalized to β-actin levels. Image study was made using Image Quant TL software, v2003 (Image Lab™ Software, Version 6.0, Bio-Rad, Laboratories, Inc., U.S., Canada).

### 2.12. Statistical Evaluation 

All the values observed in the figures and in the text are expressed as mean ± standard error and refer to at least three experiments carried out at different times. Data analysis was performed by a one-way study of variance followed by a post-hoc Bonferroni test for multiple comparisons. A *p*-value of minus than 0.05 was considered significant.

## 3. Results

### 3.1. Effect of H. erinaceus or C. versicolor, or H. erinaceus Plus C. versicolor Treatment on Histological Changes TBI-Induced

**Figure 1:**
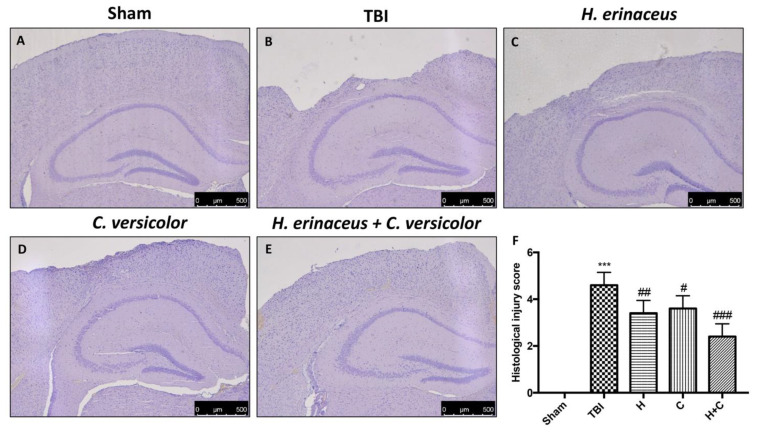
*H. erinaceus* (H), *C. versicolor* (C) and *H. erinaceus* + *C. versicolor* (H + C) reduce the severity of brain trauma**.** No alterations in brain tissues from Sham mice (**A**). In contrast, brain images after traumatic brain injury (TBI) show tissue disorganization and white matter alteration at 30 days post injury (**B**). Significant protection from TBI was evident in tissue collected from mice treated with H (**C**), C (**D**) and especially H + C (**E**). The figures are representative of at least three experiments performed on different experimental days. Each data is expressed as the mean ± standard error of the mean (SEM) of *n* = 5 male CD1 mice for each group. (**F**) *** *p* < 0.001 vs. Sham; ## *p* < 0.01 vs. TBI; # *p* < 0.05 vs. TBI; ### *p* < 0.001 vs. TBI.

Histological examination of the brain collected by TBI animals, at 30 days post-impact, showed significant tissue alteration in the perilesional area of the cortex (Figure 1B, see histological score Figure 1F) compared to Sham mice (Figure 1A, see histological score Figure 1F). We showed that the treatment with H or C reduced the degree of brain injury (Figure 1C,D, see histological score Figure 1F). Effects of the combined treatment with H and C have been shown to have a more significant effect than the single substances (Figure 1E, see histological score Figure 1F).

**Figure 2:**
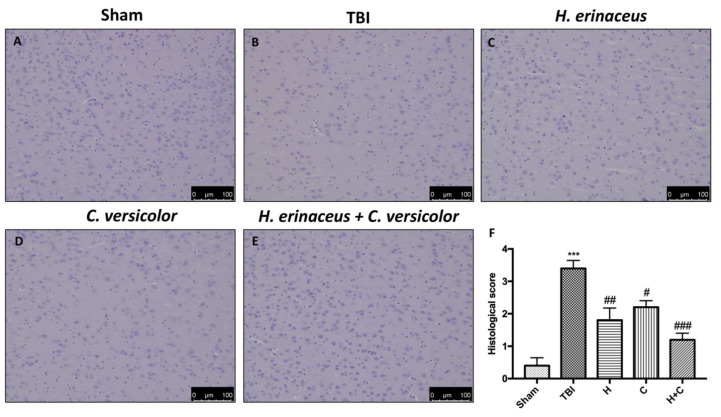
Effect of *H. erinaceus* (H), *C. versicolor* (C) and *H. erinaceus + C. versicolor* (H + C) on histological parameters in the midbrain after chronic head injury. The Sham group showed no evidence of cell degeneration in the substantia nigra (SN) (**A**), while neuronal cell loss was observed in the animals of the TBI group (**B**). Treatment with H (**C**), C (**D**) and especially H + C (**E**) restored the architecture of the midbrain. The data are representative of at least three independent experiments and are expressed as the mean ± SEM of *n* = 5 male CD1 mice for each group. (**F**) *** *p* < 0.001 with respect to Sham; ## *p* < 0.01 vs. TBI; # *p* < 0.05 with respect to TBI; ### *p* < 0.001 compared to TBI.

In addition, H&E staining was performed to evaluate the histopathological alteration induced by TBI in midbrain region. The Sham mice showed a normal brain architecture and a normal number of neurons in the midbrain (Figure 2A, see histological score Figure 2F). Instead, mice of TBI group showed evident alterations, such as cytoplasmic vacuolization and nigrostriatal neuronal cell loss (Figure 2B, see histological score Figure 2F). In contrast, mice treated with H, C and especially H + C showed a marked reduction in cytoplasmic vacuolization and cell loss in the midbrain (Figure 2C–E, see histological score Figure 2F).

### 3.2. Effect of H. erinaceus or C. versicolor, or H. erinaceus Plus C. versicolor Treatment on Inflammatory Proteins Expression in the Chronic TBI

**Figure 3:**
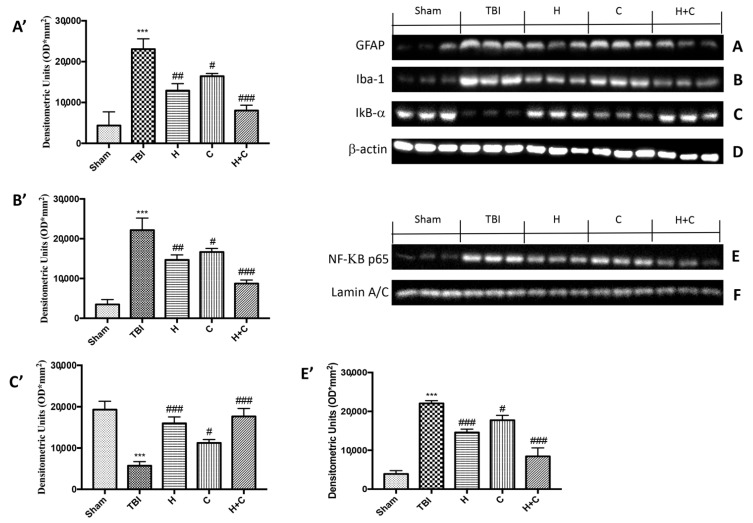
Effect of *H. erinaceus* or *C. versicolor*, or *H. erinaceus + C. versicolor* treatment on glial fibrillary acidic protein (GFAP), Iba-1, IκB-α and NF-κB p65 proteins after chronic TBI. Levels of GFAP (**A**) and Iba-1 (**B**) in midbrain region were significantly increased in the TBI group compared to Sham animals. Treatment with H, C and mostly H + C significantly reduced the activation of astrocytes (**A**,**A’**) and microglia (**B**,**B’**). Basal levels of IkB-α were found in the tissues of the Sham group; these levels were significantly reduced in the TBI mice samples (**C**,**C’**). The administration of H, C and especially H + C significantly reduced the degradation of IkB-α (**C**,**C’**). NF-kB levels were significantly increased in the nuclear fraction of the animals of the TBI group compared to the Sham animals (**E**,**E’**). H or C or H + C treatments significantly reduced nuclear factor kappa-light-chain-enhancer of activated B cells (NF-kB) translocation (**E**,**E’**). Protein lysates were also incubated with β-actin antibody (**D**) or laminin antibody (**F**) in order to verify that all samples had been loaded in equal quantities. Data are expressed as mean SEM from *n* = 5 mice/group. Densitometry analysis: (**A’**) *** *p* < 0.001 vs. Sham; ## *p* < 0.01 vs. TBI; # *p* < 0.05 vs. TBI; ### *p* < 0.001 vs. TBI; (**B’**) *** *p* < 0.001 vs. Sham; ## *p* < 0.01 vs. TBI; # *p* < 0.05 vs. TBI; ### *p* < 0.001 vs. TBI; (**C’**) *** *p* < 0.001 vs. Sham; # *p* < 0.05 vs. TBI; ### *p* < 0.001 vs. TBI; (**E’**) *** *p* < 0.001 vs. Sham; ### *p* < 0.001 vs. TBI; # *p* < 0.05 vs. TBI; ### *p* < 0.001 vs. TBI.

To study the activation of astrocytes and microglia in relation to chronic TBI and PD pathology, Western blot analysis was applied to quantify the expression of GFAP and Iba-1, markers of astrocytes and microglia, respectively. Levels of GFAP and Iba-1 were very low in the Sham group, while they were significantly elevated 30 days after TBI. Under these conditions, treatment with H or C and even more with the combination significantly reduced the increase in GFAP and Iba-1 expression in the midbrain (Figure 3A,B, see relative densitometry analysis Figure 3A′,B′). 

Furthermore, in order to evaluate the mechanism by which H or C or the combination attenuated the neuroinflammatory processes that led to the development of PD, we studied the expression of NF-κB p65 and IκB-α by Western blot analysis. The results obtained showed a basal expression of IκB-α in the control group, while a significant degradation of this protein was observed after the TBI. Treatment with the individual substances and more particularly that with the association between H and C was able to reduce the cytoplasmic degradation of IκB-α (Figure 3C, see relative densitometry analysis Figure 3C′). Regarding the nuclear translocation of NF-κB p65, our results showed low levels in the Sham group, significantly increased levels 30 days after TBI, while the treatments, especially the combination, were able to reduce the nuclear translocation of NF-κB p65 (Figure 3E, see relative densitometry analysis Figure 3E′).

**Figure 4:**
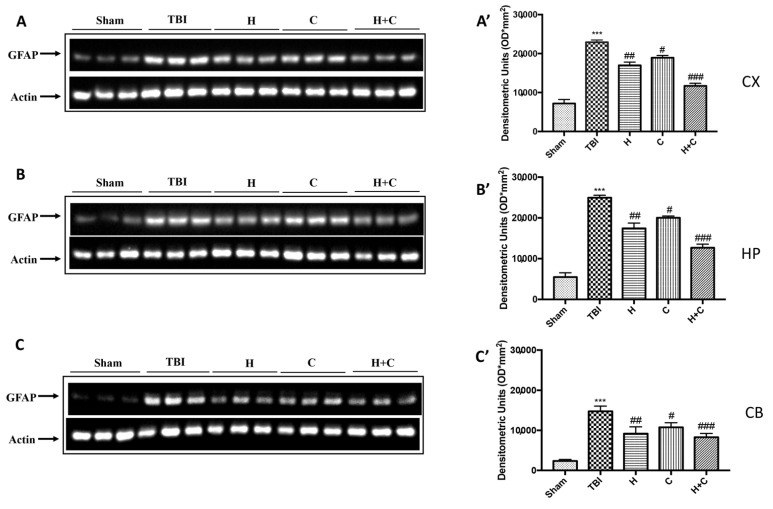
Effect of *H. erinaceus* or *C. versicolor*, or *H. erinaceus + C. versicolor* treatment on GFAP protein after chronic TBI. Western blot analysis showed that the expression of GFAP increased significantly in the TBI group in CX (**A**), HP (**B**) and CB (**C**) regions. H, C, and H + C treatment further significantly decreased the expression of GFAP in CX and HP regions (**A**,**B**); while in CB only H and H + C significantly decreased the expression of GFAP. The data are expressed as the mean SEM from n = 5 mice/group. Densitometry analysis: (**A’**) *** *p* < 0.001 vs. Sham; ## *p* < 0.01 vs. TBI; # *p* < 0.05 vs. TBI; ### *p* < 0.001 vs. TBI; (**B’**) *** *p* < 0.001 vs. Sham; ## *p* < 0.01 vs. TBI; # *p* < 0.05 vs. TBI; ### *p* < 0.001 vs. TBI; (**C’**) *** *p* < 0.001 vs. Sham; ## *p* < 0.01 vs. TBI; # *p* < 0.05 vs. TBI; ### *p* < 0.001 vs. TBI.

**Figure 5:**
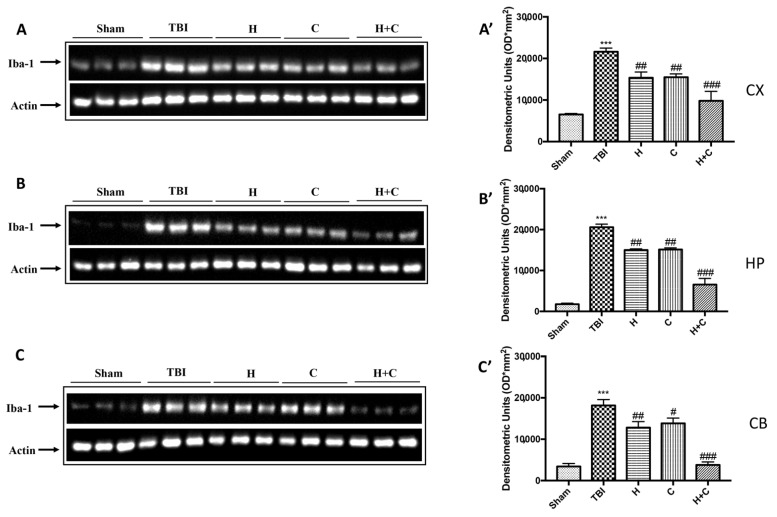
Effect of *H. erinaceus* or *C. versicolor*, or *H. erinaceus + C. versicolor* treatment on Iba-1 protein after chronic TBI. Western blot analysis showed that the expression of Iba-1 increased insignificantly in the TBI group in CX (**A**), HP (**B**) and CB (**C**) regions. H, C, and H + C treatment further significantly decreased the expression of Iba-1 (**A**–**C**). The data are expressed as the mean SEM from *n* = 5 mice/group. Densitometry analysis: (**A’**) *** *p* < 0.001 vs. Sham; ## *p* < 0.01 vs. TBI; ### *p* < 0.001 vs. TBI; (**B’**) *** *p* < 0.001 vs. Sham; ## *p* < 0.01 vs. TBI; ### *p* < 0.001 vs. TBI; (**C’**) *** *p* < 0.001 vs. Sham; ## *p* < 0.01 vs. TBI; # *p* < 0.05 vs. TBI; ### *p* < 0.001 vs. TBI.

Furthermore, the levels of GFAP, Iba-1 and IkB-α were measured by Western blot analysis in the cortex (Cx), in the hippocampus (Hp) and in the cerebellum (Cb). A significant increase of GFAP and Iba-1 was observed in the TBI group, while in animals treated with H and C and especially with H + C, a significant decrease was observed for GFAP and Iba-1 in all regions of the brain examined (Figure 4A,A’–C,C’ for GFAP and Figure 5A,A’–C,C’ for Iba-1).

**Figure 6:**
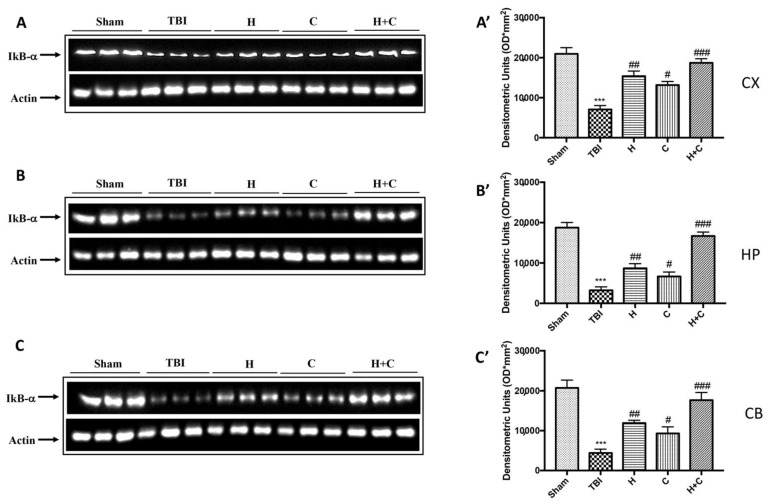
Effect of *H. erinaceus* or *C. versicolor*, or *H. erinaceus + C. versicolor* treatment on IκB-α protein after chronic TBI. Western blot analysis showed that the expression of IκB-α decreased insignificantly in the TBI group in CX (**A**), HP (**B**) and CB (**C**) regions. H, C, and H + C treatment further significantly increased the expression of IκB-α (**A**–**C**). The data are expressed as the mean SEM from *n* = 5 mice/group. Densitometry analysis: (**A’**) *** *p* < 0.001 vs. Sham; ## *p* < 0.01 vs. TBI; # *p* < 0.05 vs. TBI; ### *p* < 0.001 vs. TBI; (**B’**) *** *p* < 0.001 vs. Sham; ## *p* < 0.01 vs. TBI; # *p* < 0.05 vs. TBI; ### *p* < 0.001 vs. TBI; (**C’**) *** *p* < 0.001 vs. Sham; ## *p* < 0.01 vs. TBI; # *p* < 0.05 vs. TBI; ### *p* < 0.001 vs. TBI.

As for IkB-α, a significant decrease in the expression of this protein was observed in all brain areas examined in the TBI group, while treatment with H, C, and especially with H + C restored IkB-α levels (Figure 6A–C, see relative densitometry analysis Figure 6A’–C’). 

### 3.3. Effect of H. erinaceus or C. versicolor, or H. erinaceus Plus C. versicolor Treatment on Cellular Stress Response after Chronic TBI

Oxidative stress in the brain that activates following TBI is associated with increased expression of genes that contribute to the elimination of free radicals, the recovery of mitochondrial function, and genes sensitive to cell survival stress known as vitagenes [38]. In this regard we wanted to evaluate the effect of H or C or H + C on Nrf2/HO-1 pathway.

**Figure 7:**
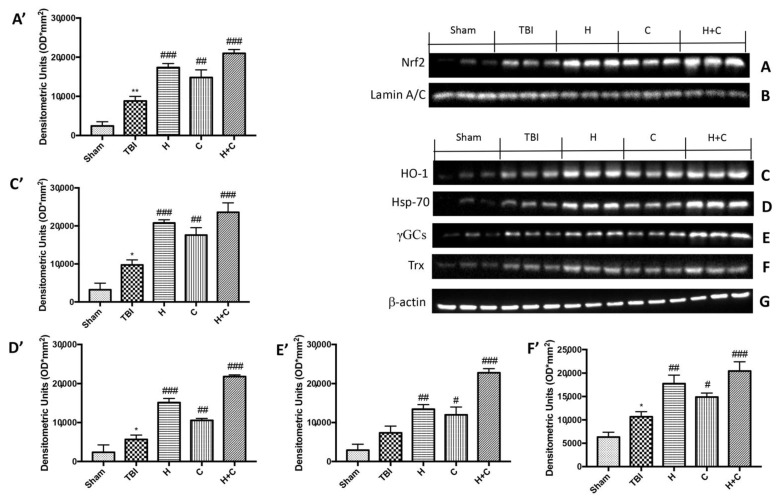
Effect of *H. erinaceus* or *C. versicolor*, or *H. erinaceus + C. versicolor* treatment on Nrf2, HO-1, Hsp-70, γGCs and Trx proteins after chronic TBI. Western blot analysis on midbrain showed that Nrf2 expression was significantly increased in the TBI group, while treatments with H, C or H + C further increased the expression of Nrf2 (**A**). HO-1 expression was increased after TBI; treatments with H, C or H + C continued to increase levels of this protein (**C**). Hsp-70 expression was increased after rotenone intoxication; treatments with H, C and especially with H + C kept the levels of this protein high (**D**). The expression of γGCs was increased in the TBI group, while treatments with H, C or H + C further increased the expression of γGCs (**E**). Finally, Trx expression was increased after TBI; treatments with H, C or H + C further increased the levels of this protein (**F**). The protein lysates were also incubated with anti-actin antibody (**G**) or laminin antibody (**B**) to verify that the quantities of the loaded samples were the same. Data are expressed as mean SEM from n = 5 mice/group. Densitometry analysis: (**A’**) ** *p* < 0.01 vs. Sham; ### *p* < 0.001 vs. TBI; ## *p* < 0.01 vs. TBI; ### *p* < 0.001 vs. TBI; (**C’**) * *p* < 0.05 vs. Sham; ### *p* < 0.001 vs. TBI; ## *p* < 0.01 vs. TBI; ### *p* < 0.001 vs. TBI; (**D’**) * *p* < 0.05 vs. Sham; ### *p* < 0.001 vs. TBI; ## *p* < 0.01 vs. TBI; ### *p* < 0.001 vs. TBI; (**E’**) ## *p* < 0.01 vs. TBI; # *p* < 0.05 vs. TBI; ### *p* < 0.001 vs. TBI; (**F’**) * *p* < 0.05 vs. Sham; ## *p* < 0.01 vs. TBI; # *p* < 0.05 vs. TBI; ### *p* < 0.001 vs. TBI.

Our results showed that the proteins responsible for anti-oxidant defense such as Nrf2, HO-1, Hsp-70, γGCs and Trx already increased after 30 days from the TBI compared to the control group. The three treatments, especially the association between H and C, was able to further and significantly increase the expression of these proteins (Figure 7A,C–F, see relative densitometry analysis Figure 7A’,C’–F’). 

**Figure 8:**
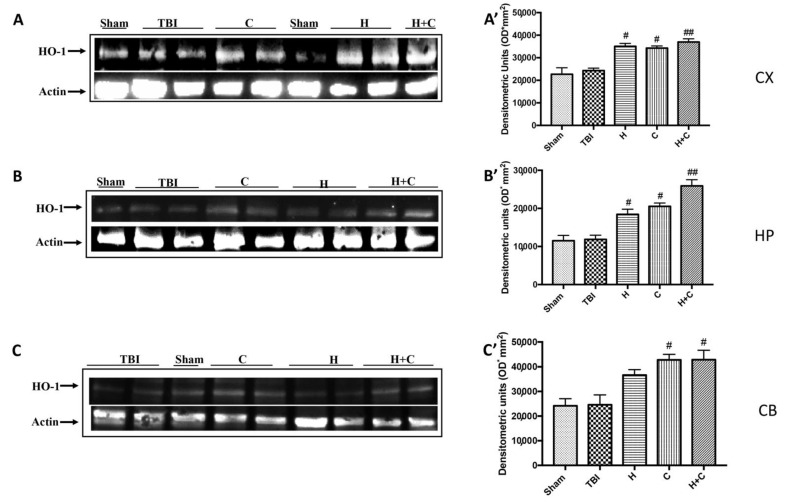
Effect of *H. erinaceus* or *C. versicolor*, or *H. erinaceus + C. versicolor* treatment on HO-1 protein after chronic TBI. Western blot analysis showed that the expression of HO-1 increased insignificantly in the TBI group in CX (**A**), HP (**B**) and CB (**C**) regions. H, C, and H + C treatment further significantly increased the expression of HO-1 (**A**–**C**). The data are expressed as the mean SEM from *n* = 5 mice/group. Densitometry analysis: (**A’**) # *p* < 0.05 vs. TBI; # *p* < 0.05 vs. TBI; ## *p* < 0.01 vs. TBI; (**B’**) # *p* < 0.05 vs. TBI; # *p* < 0.05 vs. TBI; ## *p* < 0.01 vs. TBI; (**C’**) # *p* < 0.05 vs. TBI; # *p* < 0.05 vs. TBI.

Furthermore, we investigated the effect of chronic TBI and treatment with H, C or H + C in different areas of the brain. In particular, the levels of HO-1, Hsp70, γGCs and Trx, were measured by Western blot analysis, in the cortex (Cx), in the hippocampus (Hp) and in the cerebellum (Cb). In animals treated with H and C and especially with H + C, a significant increase of HO-1 was observed in all regions of the brain examined, while this significant increase was not observed in the TBI group. Basal levels were observed in the control animals (Figure 8A–C, see relative densitometry analysis Figure 8A’–C’). 

**Figure 9:**
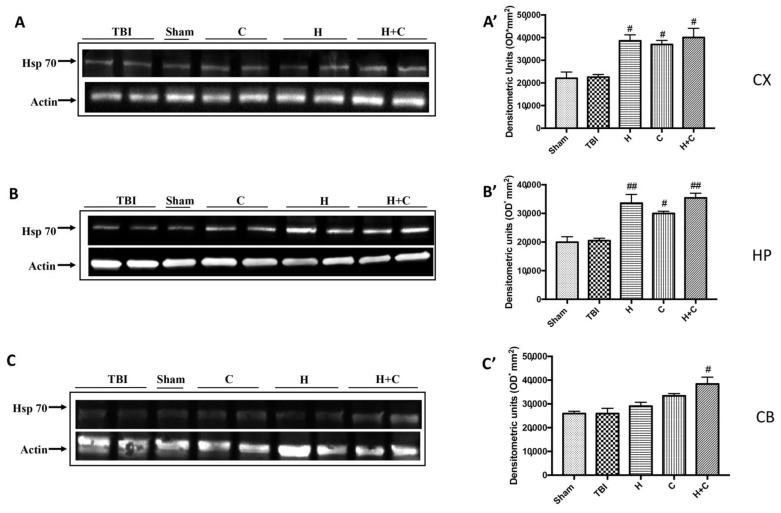
Effect of *H. erinaceus* or *C. versicolor*, or *H. erinaceus + C. versicolor* treatment on Hsp-70 protein after chronic TBI. Western blot analysis showed that the expression of Hsp-70 increased insignificantly in the TBI group in CX (**A**), HP (**B**) and CB (**C**) regions. H, C, and H + C treatment further significantly increased the expression of Hsp-70 in CX and HP regions (**A**,**B**). In CB area only H + C treatment significantly increased Hsp-70 expression (**C**). The data are expressed as the mean SEM from n = 5 mice/group. Densitometry analysis: (**A’**) # *p* < 0.05 vs. TBI; # *p* < 0.05 vs. TBI; # *p* < 0.05 vs. TBI; (**B’**) ## *p* < 0.01 vs. TBI; # *p* < 0.05 vs. TBI; ## *p* < 0.01 vs. TBI; (**C’**) # *p* < 0.05 vs. TBI.

The same trend was observed for Hsp-70. Except at the level of Cb only the H + C treatment has been shown to significantly increase the expression of this protein (Figure 9A–C, see relative densitometry analysis Figure 9A’–C’). 

**Figure 10:**
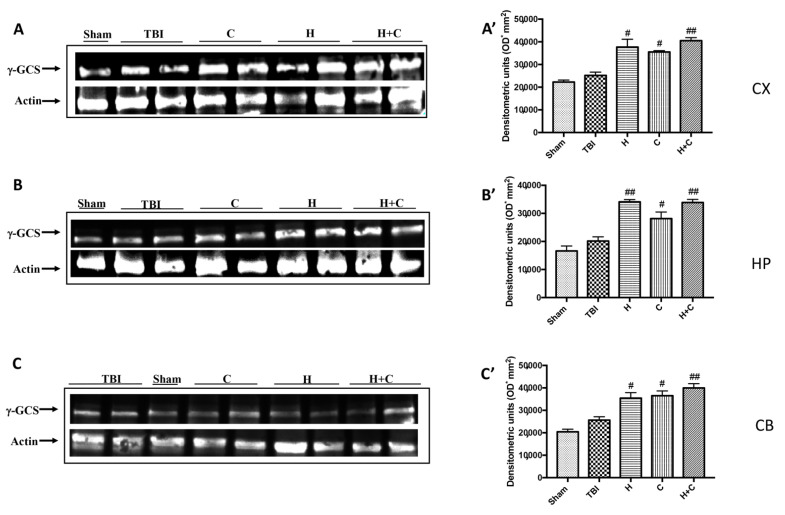
Effect of *H. erinaceus* or *C. versicolor*, or *H. erinaceus + C. versicolor* treatment on γ-GCS protein after chronic TBI. Western blot analysis showed that the expression of γ-GCS increased insignificantly in the TBI group in CX (**A**), HP (**B**) and CB (**C**) regions. H, C, and H + C treatment further significantly increased the expression of γ-GCS in CX, HP and CB regions (**A**–**C**). The data are expressed as the mean SEM from n = 5 mice/group. Densitometry analysis: (**A’**) # *p* < 0.05 vs. TBI; # *p* < 0.05 vs. TBI; ## *p* < 0.01 vs. TBI; (**B’**) ## *p* < 0.01 vs. TBI; # *p* < 0.05 vs. TBI; ## *p* < 0.01 vs. TBI; (**C’**) # *p* < 0.05 vs. TBI; # *p* < 0.05 vs. TBI; ## *p* < 0.01 vs. TBI.

Also, with regard to γ-GCs, a significant increase in the expression of this protein was observed by treatments with H, C and H + C in all brain areas examined, compared to the Sham and TBI group (Figure 10A–C, see relative densitometry analysis Figure 10A’–C’). 

**Figure 11:**
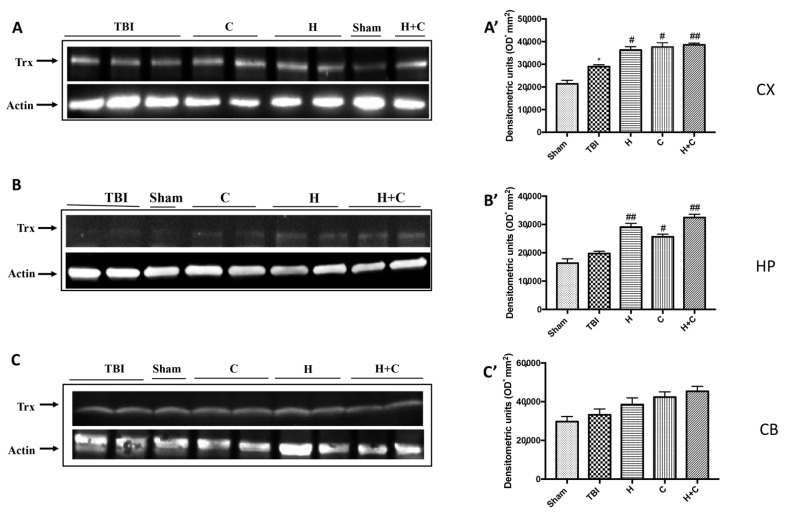
Effect of *H. erinaceus* or *C. versicolor*, or *H. erinaceus + C. versicolor* treatment on Trx protein after chronic TBI. Western blot analysis showed that the expression of Trx increased insignificantly in the TBI group in HP (**B**) and CB (**C**) regions and significantly in CX (**A**). H, C, and H + C treatment further significantly increased the expression of Trx in CX and HP regions but not in CB (**A**–**C**). The data are expressed as the mean SEM from *n* = 5 mice/group. Densitometry analysis: (**A’**) * *p* < 0.05 vs. Sham; # *p* < 0.05 vs. TBI; # *p* < 0.05 vs. TBI; ## *p* < 0.01 vs. TBI; (**B’**) ## *p* < 0.01 vs. TBI; # *p* < 0.05 vs. TBI; ## *p* < 0.01 vs. TBI; (**C’**) not significant.

Finally, the expression of the Trx protein was significantly increased by treatments with H, C and H + C only in Cx and Hp. No significant increase was observed at the Cb level (Figure 11A–C, see relative densitometry analysis Figure 11A’–C’).

### 3.4. Effect of H. erinaceus or C. versicolor, or H. erinaceus Plus C. versicolor Treatment on Changes of PD Markers and on the Apoptotic Process after Chronic TBI 

**Figure 12:**
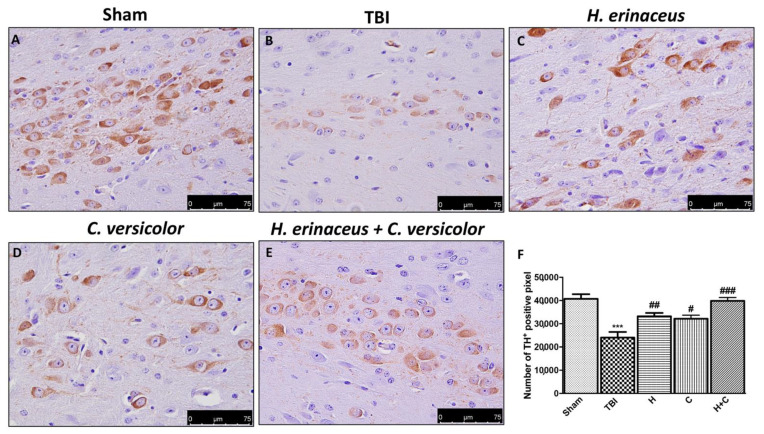
Effect of *H. erinaceus* or *C. versicolor*, or *H. erinaceus + C. versicolor* treatment on TH expression after chronic TBI. Immunohistochemical analysis on midbrain region showed a significant loss of TH-positive cells (**B**) compared to Sham mice (**A**). Animals treated with H, C and especially with H + C revealed an increase in the expression of TH (**C**–**E**). Data is expressed as % TH-positive pixels and are the SEM means of *n* = 5 mice/group. (**F**) *** *p* < 0.001 vs. Sham; ## *p* < 0.01 vs. TBI; # *p* < 0.05 vs. TBI; ### *p* < 0.001 vs. TBI.

**Figure 13:**
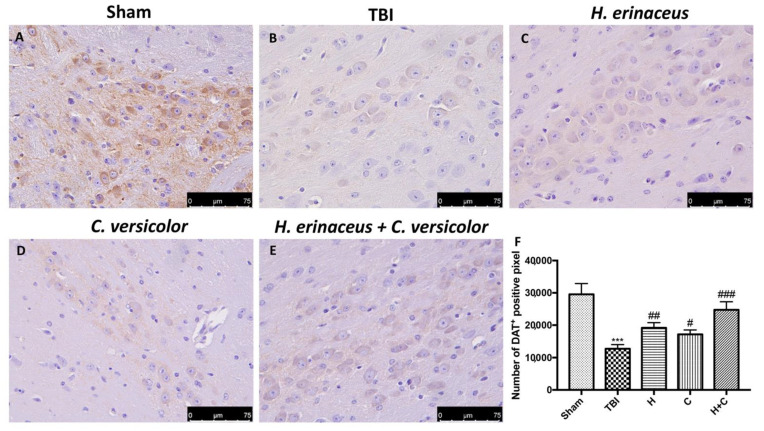
Effect of *H. erinaceus* or *C. versicolor*, or *H. erinaceus + C. versicolor* treatment on DAT expression after chronic TBI. Immunohistochemical analysis on midbrain region showed a significant loss of DAT-positive cells (**B**) compared to Sham mice (**A**). Animals treated with H, C and especially with H + C revealed an increase in the expression of DAT (**C**–**E**). Data is expressed as % DAT-positive pixels and are the SEM means of *n* = 5 mice/group. (**F**) *** *p* < 0.001 vs. Sham; ## *p* < 0.01 vs. TBI; # *p* < 0.05 vs. TBI; ### *p* < 0.001 vs. TBI.

To observe if chronic TBI can modify PD-like markers, brain sections from mice of each groups were stained with dopaminergic-specific markers (TH and DAT). The expression of TH-positive neurons and DAT was notably decreased 30 days after TBI (Figure 12B (for TH) and Figure 13B (for DAT), respectively, see densitometric analysis, Figure 12F and Figure 13F) compared to Sham group (Figure 12A (for TH) and Figure 13A (for DAT), respectively, see densitometric analysis, Figure 12F and Figure 13F). Treatment with H or C or the combination increased the levels of these proteins. In particular, a greater efficacy of treatment with H + C was observed (Figure 12C–E (for TH) and Figure 13C–E (for DAT), respectively, see densitometric analysis, Figure 12F and Figure 13F).

**Figure 14:**
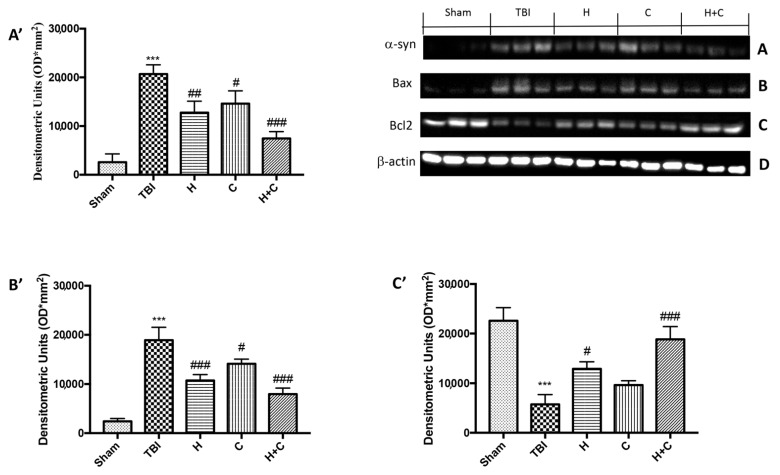
Effect of *H. erinaceus* or *C. versicolor*, or *H. erinaceus + C. versicolor* treatment on α-syn expression and apoptotic process after chronic TBI. Western blot analysis on midbrain region revealed a significant increase in α-syn in the TBI group compared to Sham animals. The H, or C or H + C treatment significantly reduced the increase in this protein (**A**). Western blot analysis demonstrated Bax expression to be significantly increased in the TBI group, whereas treatment with H, C and especially H + C significantly limited the rise in Bax expression (**B**). Finally, Bcl-2 expression was reduced after TBI; however, treatment with H, C and H + C restored the basal levels (**C**). Protein lysates were also incubated with a β-actin antibody (**D**) in order to verify that all samples had been loaded in equal quantities. The data are expressed as the mean SEM from *n* = 5 mice/group. Densitometry analysis: (**A’**) *** *p* < 0.001 vs. Sham; ## *p* < 0.01 vs. TBI; # *p* < 0.05 vs. TBI; ### *p* < 0.001 vs. TBI; (**B’**) *** *p* < 0.001 vs. Sham; ### *p* < 0.001 vs. TBI; # *p* < 0.05 vs. TBI; (**C’**) *** *p* < 0.001 vs. Sham; # *p* < 0.05 vs. TBI; ### *p* < 0.001 vs. TBI.

On the contrary, by Western blot analysis we observed that chronic TBI caused an evident increase in α-syn compared to the Sham group, while the treatment with the single compounds reduced α-syn in the brain but the association acted more on the prevention of the accumulation of this protein (Figure 14A, see relative densitometry analysis Figure 14A’).

In order to investigate the effect of H or C or the association H + C on apoptotic events induced by chronic TBI we assessed, by Western blot analysis, the expression of pro-apoptotic protein such as Bax. The results obtained displayed an important increase of Bax levels after TBI respect the Sham group, while a reduction of expression of Bax was evident after H or C treatment, especially after the administration of H + C (Figure 14B, see relative densitometry analysis Figure 14B’). Moreover, we analyzed the anti-apoptotic factor Bcl-2 and we observed ah high level of Bcl-2 in the Sham group and an important reduction in TBI group; instead treatments with H, C and especially with the association significantly restored Bcl-2 expression (Figure 14C, see relative densitometry analysis Figure 14C’).

### 3.5. Effect of H. erinaceus or C. versicolor, or H. erinaceus Plus C. versicolor Treatment on Depression- and Anxiety-Like Behaviors in the Mouse after Chronic TBI 

**Figure 15:**
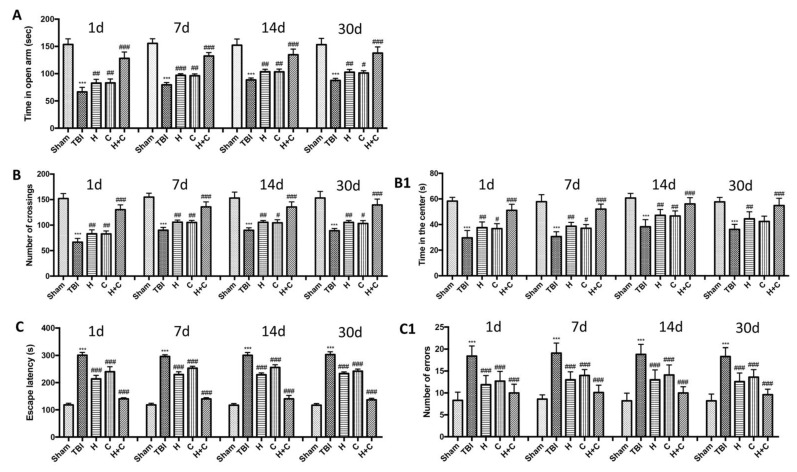
Effect of *H. erinaceus* or *C. versicolor*, or *H. erinaceus + C. versicolor* treatment on anxious behavior after chronic TBI. The degree of non-motor impairment was assessed blindly at different time points (1, 7, 14 and 30 days) and with different behavioral tests. Elevated plus maze (EPM) testing showed that TBI animals spent less time exploring open arms than Sham animals. While the animals treated with H, C and especially with H + C had a greater propensity to remain in the open arms (**A**). OF tests showed that TBI mice traveled significantly shorter distances and did not stay in the central area longer, unlike treated mice which instead moved longer distances (**B**,**B1**). Barnes maze test revealed a decrease in TBI-induced spatial learning by increasing the latency for escape and mean number errors, which were reduced by all three treatments and in particular by the association (**C**,**C1**). Data are means ± SEM of *n* = 10 mice/group. (**A**) *** *p* < 0.001 vs. Sham; # *p* < 0.05 vs. TBI; ## *p* < 0.01 vs. TBI; ### *p* < 0.001 vs. TBI; (**B**,**B1**) *** *p* <0.001 vs. Sham; # *p* < 0.05 vs. TBI; ## *p* < 0.01 vs. TBI; ### *p* < 0.001 vs. TBI; (**C**,**C1**) *** *p* < 0.001 vs. Sham; ### *p* < 0.001 vs. TBI.

Using various behavioral tests, such as the EPM, open field test and Barnes maze, we compared the degree of anxiety and locomotor function of mice undergoing chronic TBI with control mice and treatment mice at different days (1, 7, 14, and 30 days). In the EPM test we observed that TBI mice spent less time in open arms than control mice, while treated mice tended to spend more time in open arms especially those treated with H + C (Figure 15A). This state of anxiety observed in mice with the lesion was also confirmed by the open field test. Mice with TBI showed an increase in thigmotaxis, i.e., a tendency to stay outside the field near the wall, compared to Sham mice. The treated animals instead showed a shorter time spent in the center of the open field (Figure 15B) and shorter distance covered in the center of the open field (Figure 15(B1)). Furthermore, cognitive dysfunctions such as spatial learning and memory were assessed using the Barnes maze. The results obtained showed a significant reduction in spatial learning and an increase in escape latency and in the average number of errors in the mice of the TBI group for all observed times, compared to the Sham group. The animals treated with H, or C or H + C had a similar behavior to the Sham group, in fact they were shown to quickly learn to escape the open field and reach the black escape box, having a significant reduction in escape latency (Figure 15C,C1).

### 3.6. Characterization of H. erinaceus and C. versicolor

**Figure 16:**
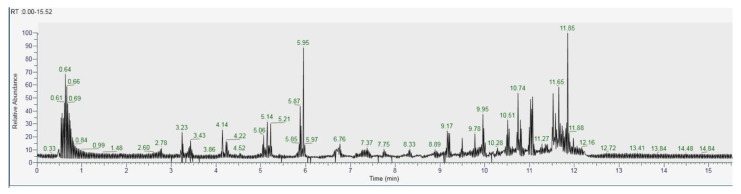
GC-MS/MS chromatogram of *Coriolus versicolor.*

**Figure 17:**
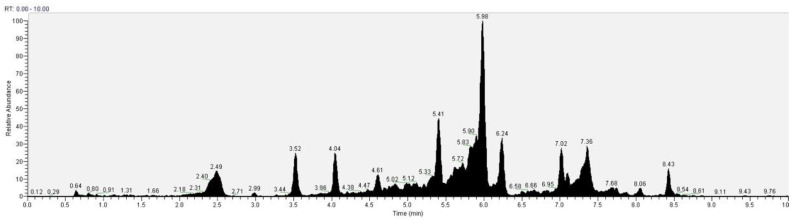
LC–Orbitrap-MS of *Coriolus versicolor.*

The recognition of the analytes was conducted both with the analysis of single standards, the method of exact masses, convolutions with algorithms and NIST library. The relative abundance refers to the ratio of the area of only the peaks recognized with certainty. As for the analysis of *C. versicolor* in GC, the most commonly found compounds are hexadecenoic acid (15.14), glucose (14.04%) and hexadecane (13.59%) (Figure 16, Table 1). In the LC analysis, the largest relative abundance found is vanillic acid (11.93%). Relevant are the abundances of ferulic acid (10.03%), apigenin (9.34%), catechin, epicatechin (7%), quercetin (6.95) and kaempferol (6.24%). Luteolin, gallic acid and syringic acid show 2.08% 3.61% and 3.75%, respectively. The only compounds found in both GC and LC are kaempferol with relative abundances 11.54% in GC and 6.24% in LC and vanillic acid with relative abundances 7.75% in GC and 11, 93% in LC. The other compounds found in GC are fatty acids, and sphingolipids (Figure 17, Table 2). 

**Figure 18:**
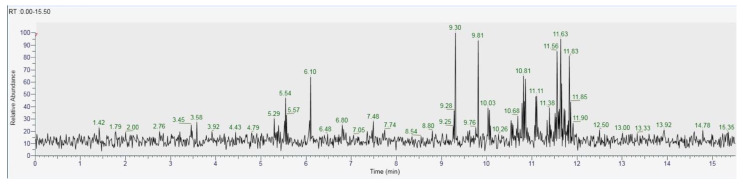
GC-MS/MS chromatogram of *Hericium erinaceus.*

**Figure 19:**
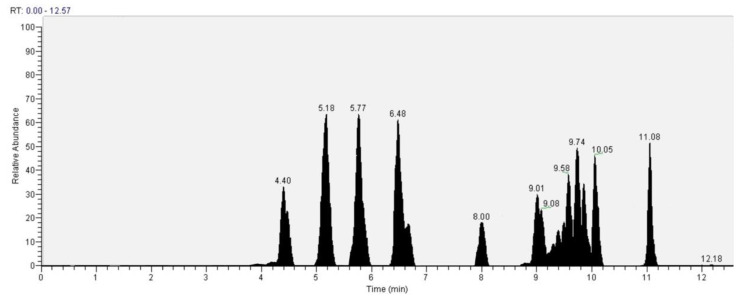
LC–Orbitrap-MS of *Hericium erinaceus.*

As for the GC analysis of *H. erinaceus*, ergosterol (15.34%), ergothioneine (12.72%), coumaric acid (11.03%), glucose (14,05%) and tartaric acid (10.45%) are the most representative compounds (Figure 18, Table 3). In the LC analyses compounds with high relative abundance values compounds such as catechin (14.01%), epicatechin (13.46%) vanillic acid (11.07), kaempferol (9.73%), quercetin (8.31%) and cinnamic acid (8.05%) were recognized. The other compounds found by GC are fatty acids. The compounds found in both GC and LC are coumaric acid with relative abundances 11.03% in GC and 3.38% in LC, kaempferol 1.86% in GC and 9.73% in LC and vanillic acid with relative abundances 4.22% in GC and 11.03% in the LC analysis (Figure 19, Table 4).

## 4. Discussion

The study of natural compounds is of worldwide interest due to their numerous biological activities. Many sources of these compounds including *H. erinaceus* and *C. versicolor* mushrooms have been shown to have antioxidant and anti-inflammatory power, especially in diseases affecting the nervous system. In particular, their beneficial power was observed in AD [39]. On the basis of the data in the literature, our hypothesis was that *H. erinaceus* and *C. versicolor* were able to prevent the propagation of secondary damage in remote areas with respect to the primary region of the lesion in the advanced stage of TBI and therefore to slow down or inhibit the progression of the neurodegenerative processes leading to PD. Several studies have validated that in animal models of TBI there is the appearance of pathophysiological processes characteristic of PD [6,40,41]. Impellizzeri et al. showed that the TBI induced in mice following controlled cortical impact causes a significant reduction in PD specific markers such as TH and DAT after 30 days. They also observed an important increase in inflammatory markers such as GFAP, Iba-1 and the degradation of Ikb-α which confirm the importance of neuroinflammation in neurodegenerative processes [6]. Our histopathological results confirmed the connection between TBI and alterations in the midbrain, the region most involved in PD. In particular, 30 days after the induction of TBI, an alteration in the normal architecture of the brain and in the number of neurons in the substantia nigra was observed; while a potential beneficial effect was observed in animals treated with H, C and especially with H + C. After these first analyzes, we investigated how these compounds acted at the molecular level. Known that the alterations in astrocytes and microglia are present in patients with PD and that these cell populations remain activated even after 30 days from TBI [42], we have shown that treatment with H, C and especially with their combination was able to reduce the activation of both GFAP and Iba-1 in the midbrain. The activation of astrocytes and microglia is connected to the activation of the NF-κB pathway. It has been observed that the activation of this pathway persists for at least one year after the cortical lesion [43]. For this reason we can suppose that the NF-κB pathway plays a key role in long-term inflammatory processes following TBI that can lead to neurodegeneration. Our results demonstrate the ability of H and C, administered individually or in combination, to counteract the activation of the pathway and therefore to reduce the degradation of IKB-α and the nuclear translocation of NF-κB. The interruption of homeostasis caused by TBI not only induces the cascade of inflammatory signals but is also responsible for the production of reactive oxygen species [8,9,38,44]. Therefore, we believe it is important to combat not only inflammatory processes but also the oxidative stress that is activated following TBI and that can spread to regions far from the impact area such as the midbrain where it can contribute to the progression of neurodegenerative phenomena. Our results highlighted an increase in the expression of proteins responsible for the antioxidant defense system such as Nrf-2, HO-1, Hsp-70, γGCs and Trx at the midbrain level and the ability of H, C and H + C to act on these systems by further activating them and thus allowing to restore the normal physiological balance between production and elimination of oxidant species necessary to reduce the oxidative stress of brain cells. An additional study that we wanted to perform was to evaluate both neuroinflammation and oxidative stress in other brain regions such as the cortex, hippocampus and cerebellum. Our results demonstrated that oxidative stress continued to be active in the previously indicated regions even 30 days after TBI. However, this activation was not as significant as in the midbrain where neurodegenerative processes probably create a greater oxidative imbalance. On the other hand, we observed an activation of astrocytes and microglia also in the other regions of the brain examined. This indicates that the inflammatory processes spread from the impact region and remain active even 30 days after the TBI. However, treatments with H, C and H + C were able to activate the anti-oxidant defense systems and reduce inflammatory markers not only in the midbrain but also in the cortex, hippocampus and cerebellum. Time course studies would be interesting to evaluate how and when oxidative stress and inflammation spread from the lesion area to other areas of the brain. The inflammatory environment and oxidative imbalance that are created following TBI promote the accumulation of α-syn and the death of dopaminergic neurons in the midbrain region [45,46]. As demonstrated by our results, 30 days post-TBI animals exhibit increased α-syn levels and decreased TH and DAT markers. Furthermore, cell death in the midbrain has been demonstrated by evaluating specific proteins for apoptosis such as Bax and Bcl-2. Treatments with H, C and especially with H + C have shown their ability to prevent the accumulation of α-syn and neuronal death demonstrated by an increase in levels of TH, DAT and Bcl-2 and by a reduction in expression of Bax. Finally, we performed behavioral tests in order to demonstrate that the alterations in the midbrain also reflected behavioral alterations. PD presents not only motor symptoms, but also non-motor symptoms that can lead to cognitive and psychiatric disorders [47,48]. In terms of PD-related non-motor symptoms, chronic TBI induced anxious behavior in animals and reduced spatial learning, while treatments were able to reduce such behavioral changes. However, no significant variations were observed between the different times in which the behavioral tests were performed.

## 5. Conclusions

In conclusion, our discovery opens up potential neuroprotective strategies since for the first time it has been shown that the mushrooms *H. erinaceus* and *C. versicolor* and in particular their combination are able to act on specific molecular mechanisms that link chronic TBI and PD. In particular, they seem to act on microglia and consequently on the NF-κB pathway, reducing the neuroinflammation that spreads from the cortex to other areas of the brain. Furthermore, these compounds act on oxidative stress which also spreads to the midbrain thus limiting the accumulation of α-syn and the development of PD. Therefore, *H. erinaceus* and *C. versicolor* could be used as nutritional products for the prevention of neurodegenerative processes related to chronic TBI. However, further studies will be needed to investigate which of the various compounds characterized in these two mushrooms are most implicated in this neuroprotective role.

## Figures and Tables

**Table 1:** GC-MS/MS analysis of *Coriolus versicolor.*

Compound	Retention Time (min)	Area (%)
Hexadecanoic acid	0.64	15.14
Glycerol	2.78	0.76
Myristic acid	3.23	2.79
Palmitic acid	3.43	1.01
Stearic acid	4.14	2.84
Tetradecane	4.22	0.97
3-Methoxy-4-benzaldehyde	4.52	0.4
Sphinganine	5.06	1.02
8-Sphingenine	5.14	2.89
4-Hydroxysphinganine	5.21	2.76
α-Cadinene	5.87	5.05
Hexadecane	5.95	13.59
Oxybenzaldehyde	6.76	0.97
Homovanillyl alcohol	7.37	0.61
Vanillic acid	7.75	0.42
Octadecane	9.17	3.38
Oleic Acid	9.50	1.84
Linoleic acid	9.78	2.51
3,5-Dimethoxy-4-benzoate	9.95	3.61
(1-Methyldodecyl)-benzene	10.51	2.91
9-Dodecenoic methyl ester	11.03	4.78
Kaempferol	11.54	5.32
Coumaric acid	11.57	3.46
Eicosane	11.65	6.93
Glucose	11.85	14.04

**Table 2:** LC–Orbitrap-MS analysis of *Coriolus Versicolor.*

Compound	Retention Time (min)	Area (%)	Exact Mass
Gallic acid	2.49	3.61	169.014
Caffeic acid	2.99	0.79	179.034
Catechin	3.52	7.73	289.071
Epicatechin	4.04	7.56	289.071
Vanillic acid	5.41	11.93	167.041
Syringic acid	5.72	3.75	197.045
Hydroxybenzoic acid	5.98	29.84	138.03
Ferulic acid	6.24	10.03	193.057
Naringenin	6.5	0.88	272.067
Rutin	6.66	1.35	610.012
Quercetin	7.02	6.95	447.093
Apigenin	7.36	9.34	271.060
Luteolin	8.06	2.08	286.04
Kaempferol	8.43	6.24	285.040

**Table 3:** GC-MS/MS analysis of *Hericium erinaceus.*

Compound	Retention Time (min)	Area (%)
Hexadecenoic acid	3.45	0.74
Palmitic acid	5.29	2.05
Stearic acid	5.54	5.97
Octadecane	6.10	7.83
Ergosterol	9.30	15.34
Ergothioneine	9.81	12.72
Vanillic acid	10.03	4.22
Oleic Acid	10.81	8.73
Linoleic acid	11.11	5.01
Kaempferol	11.38	1.86
Coumaric acid	11.56	11.03
Glucose	11.63	14.05
Tartaric acid	11.83	10.45

**Table 4:** LC–Orbitrap-MS analysis of *Hericium Erinaceus.*

Compound	Retention Time (min)	Area (%)
Ascorbic acid	4.4	4.75
Caffeic acid	4.47	3.22
Catechin	5.18	14.01
Epicatechin	5.77	13.46
Vanillic acid	6.48	11.07
Chlorogenic acid	6.53	2.98
Hydroxybenzoic acid	8.00	3.76
Ferulic acid	9.01	5.03
*p*-Coumaric acid	9.08	3.38
Sinaptic acid	9.58	6.28
Cinnamic acid	9.74	8.05
Rutin	9.83	5.97
Quercetin	10.05	8.31
Kaempferol	11.08	9.73

## Data Availability

The datasets used in the current study are available from the corresponding author on reasonable request.
